# Three-dimensional close-to-substrate trajectories of magnetic microparticles in dynamically changing magnetic field landscapes

**DOI:** 10.1038/s41598-022-25391-z

**Published:** 2022-12-03

**Authors:** Rico Huhnstock, Meike Reginka, Claudius Sonntag, Maximilian Merkel, Kristina Dingel, Bernhard Sick, Michael Vogel, Arno Ehresmann

**Affiliations:** 1 Institute of Physics and Center for Interdisciplinary Nanostructure Science and Technology (CINSaT), University of Kassel, Heinrich-Plett-Strasse 40, 34132 Kassel, Germany; 2 Artificial Intelligence Methods for Experiment Design (AIM-ED), Joint Lab of Helmholtzzentrum für Materialien und Energie, Berlin (HZB) and University of Kassel, Hahn-Meitner-Platz 1, 14109 Berlin, Germany; 3 Intelligent Embedded Systems, University of Kassel, Wilhelmshöher Allee 71-73, 34121 Kassel, Germany; 4 Present Address: Institute for Materials Science, Kiel University, Kaiserstraße 2, 24143 Kiel, Germany

**Keywords:** Applied physics, Colloids, Biosensors, Magnetic properties and materials

## Abstract

The transport of magnetic particles (MPs) by dynamic magnetic field landscapes (MFLs) using magnetically patterned substrates is promising for the development of Lab-on-a-chip (LOC) systems. The inherent close-to-substrate MP motion is sensitive to changing particle–substrate interactions. Thus, the detection of a modified particle–substrate separation distance caused by surface binding of an analyte is expected to be a promising probe in analytics and diagnostics. Here, we present an essential prerequisite for such an application, namely the label-free quantitative experimental determination of the three-dimensional trajectories of superparamagnetic particles (SPPs) transported by a dynamically changing MFL. The evaluation of defocused SPP images from optical bright-field microscopy revealed a “hopping”-like motion of the magnetic particles, previously predicted by theory, additionally allowing a quantification of maximum jump heights. As our findings pave the way towards precise determination of particle–substrate separations, they bear deep implications for future LOC detection schemes using only optical microscopy.

## Introduction

Lab-on-a-chip (LOC) systems are considered a possible solution for the rising demand for fast and inexpensive medical diagnostic tools^1–3^. For practical implementation, magnetic particles (MPs) of micro- and nanoscopic sizes with various shapes and compositions are discussed as central functional components^4–7^. Their surfaces can be customized by functional chemical groups specific to bind disease markers or pathogens from a screened body fluid, for instance. As controllable actuation is possible by applying magnetic fields, MPs provide several key functionalities of LOC systems: Liquid mixing^8^, biomolecule capture, analyte transport and separation, and finally detection (among others)^9,10^. To transport MPs to a designated chip area, magnetic stray fields, varying on the micrometer scale, superposed with a macroscopic dynamically changing external field have been used^11–13^. The necessary magnetic stray field landscapes (MFLs) emerge either from micro-structured magnetic elements on a substrate surface^14–17^ or from topographically flat magnetic thin films with a domain pattern^18–20^. In the latter case, the domains either form naturally (e.g., in magnetic garnet films)^18^ or are artificially imprinted into the thin film^21,22^. An outstanding advantage of the use of MFLs is a comparably large magnetic field gradient within a small volume (over small distances), allowing for rapid MP transport^19^ with local steady-state velocities of more than 100 µm/s^23,24^. It is important to note, that the motion of MPs in these transport concepts occurs close to the substrate surface. The MP motion dynamics are mainly governed by the magnetic gradient forces, the hydrodynamic drag exerted by the surrounding liquid, and the electrostatic and electrodynamic interactions with the underlying substrate^25^. If gravitation and buoyancy are neglected, the balance of magnetic, electrostatic, and van der Waals forces determines the equilibrium distance between particle and substrate, i.e., their interplay defines the position of the particles within the magnetic field landscape^23,25^, which in turn influences strongly the lateral MP motion. The experimental determination of this vertical distance would therefore be, besides the two-dimensional MP trajectories, an important additional observable for the understanding of the MP motion over a flat substrate. Moreover, resolving vertical MP positions along the lateral trajectories creates an alternative access for bio-sensing applications: The electrostatic and electrodynamic interactions between MP and substrate change upon analyte binding^23^ either on the particle’s or on the substrate’s surface when the opposing surface remains unchanged. Therefore, determining a change in the separation distance between MP and substrate upon analyte binding possesses a large potential as a bio-detection technique.

Here, we present the application of a comparably simple method and proof of concept to experimentally determine three-dimensional (3D) trajectories of superparamagnetic particles (SPPs) transported within an artificially created dynamically changing MFL above a topographically flat substrate with high temporal resolution. Changes in the SPPs' sharpness in microscope images, as they move relative to the microscope focal plane^26–28^, will be used in combination with a calibration procedure and lateral single particle tracking by machine learning aided software^29^ to determine the MPs’ 3D motion. The experimental results will be corroborated by a numerical model to estimate the separation distance change between substrate and SPP, upon changing their lateral in-plane position. The current work lays the foundation for dedicated analyte detection in LOC devices by simply observing the 3D MP motion through a microscope and for a new multiplex method of scanning material characteristics of flat or topographic magnetic surfaces, very similar to magnetic force microscopy, with many probes operated in parallel.

### Experimental concept overview

**Figure 1:**
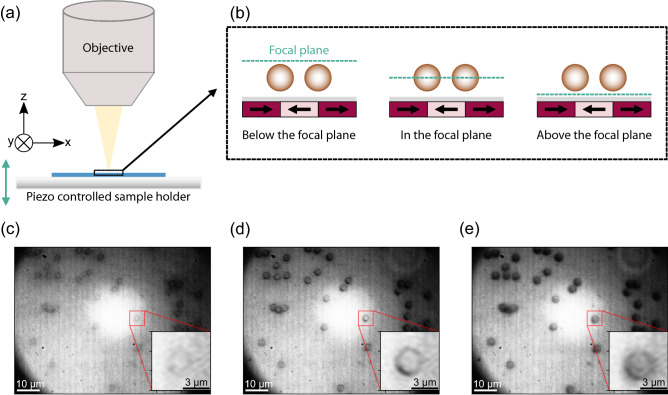
Acquisition of in and out of focus bright-field microscope images for axial position determination of SPPs. (**a**) Sketch of a focus sweep procedure. (**b**) SPPs are trapped within the local MFLs above a magnetic substrate with hh/tt magnetized stripe domains (indicated by black arrows). SPPs are moved from a position below the focal plane (**c**), through the focal plane (**d**) to a position above it (**e**). Exemplary images for micromer-M SPP (*d* = 3 µm) are shown for the three conditions. These images are used to calibrate the *z*-position of the SPP relative to the focal plane within transport experiments.

Directed SPP transport is induced in the current work by a superposition of the MFL with weak external magnetic field pulses, resulting in a periodic transformation of the particles’ potential energy. The MFL stems from a prototypical magnetic stripe domain pattern with a head-to-head (hh) and tail-to-tail (tt) magnetization configuration, engineered in an exchange-biased (EB) thin film system using ion bombardment induced magnetic patterning (IBMP)^21,30,31^. This technique allows, in general, for the fabrication of arbitrary domain patterns in EB thin films. Here, we exemplarily describe the 3D motion behavior for SPP above such a stripe pattern. The 3D tracking was achieved by implementing in- and out of focus particle image analysis in bright-field microscopy, as known from literature^26–28,32–34^. This technique has so far only seen application in the investigation of dynamic particle behavior inside confined microfluidic structures^26,28,32^ or of biological processes (in this case by mostly studying fluorescent particles)^33,35^. Specific characteristics of the particle images (e.g., diffraction rings caused by Mie scattering^36^, astigmatism in images using cylindrical lenses^37^) changing with *z*-position during a focus sweep partly in combination with cross-correlation based matching algorithms had been analyzed so far^28,32,38^. The determination of the SPPs’ axial coordinate with respect to the microscope’s focal plane has been achieved in this work by an image sharpness-based calibration procedure suitable for the studied system, experimentally realized by the setup schematically shown in Fig. 1a. This calibration has been demonstrated to work for two different types of SPPs with different optical responses. Subsequently, the “hopping” like motion behavior of SPP that is theoretically expected for the applied actuation concept^39^ could be quantitatively resolved.

## Results

### Calibration procedure for the *z*-coordinate

SPPs with sizes in a diameter range between 2.8 and 4 µm have been placed inside a microfluidic chamber on top of a magnetically patterned and topographically flat substrate. The magnetic pattern consisted of 5 µm-wide stripe domains with a periodically alternating hh/tt magnetization direction (see arrows in Fig. 1b). An additional spacer layer of Poly(methyl methacrylate) (PMMA) of 150 nm thickness has been deposited on top of the magnetic substrate to prevent SPP adsorption to the substrate surface^11,19,23^. As shown earlier, MPs are attracted to the magnetic domain walls, since they represent local minima of the MPs' potential energies^19,22,24,40,41^. Without applied external fields, the positions above the two domain wall types (hh or tt) are energetically degenerate for the SPPs. Subsequent lateral transport of SPP is achieved by applying external trapezoidal magnetic field pulses in *x*- and *z*-directions^19,22,23,42^.

MP motion has been video-recorded by an optical bright-field reflection microscope with a temporal resolution of 1000 frames per second (fps). SPPs’ *z*-positions have been determined in the individual frames by correlating image changes caused by SPP focusing/defocusing to SPP images, which are calibrated for a *z*-position relative to the focal plane of the microscope. Hence, for the present experiments, an existing setup consisting of an optical unit and electromagnets (for applying external magnetic fields)^19^ was extended by a piezo stage controlled sample holder (see Fig. 1a), which moves the sample holder vertically (*z*-direction) with a possible technical resolution of 3 nm. This way, SPPs at their equilibrium position within the MFL have been swept through the focal plane of the used 100 × magnification objective (Fig. 1b). Depending on the SPP position below the focal plane, in focus, or above the focal plane, images with different signal-to-noise ratios and particle sharpness were recorded. These images are exemplarily shown for micromer-M SPPs with a diameter of 3 µm in Fig. 1c–e.

The optical axial resolution is described by the depth of field (DOF) of the utilized microscope setup. The DOF characterizes the axial range throughout an imaged specimen, where all object planes are simultaneously in focus. In the diffraction limit, the DOF can be expressed by $\mathrm{DOF }= \frac{\lambda }{2\cdot {\mathrm{NA}}^{2}}$, where λ describes the wavelength of the used light and NA is the numerical aperture of the objective^35^. The DOF of the present setup (central wavelength λ ≈ 500 nm for white light, NA = 1.4) is estimated to be approximately 130 nm. Thus, the comparably large NA not only provides the high lateral image resolution needed for two-dimensional particle tracking but also results in a rather thin focal plane which is critical for a high resolution in determining the axial SPP position with respect to that focal plane. For a quantification of the SPPs’ *z*-distance relative to the microscope’s focal plane, a suitable metric for correlation of the particle image characteristics to axial coordinate during a focus sweep is needed. Approaches based on calculated point-spread functions for the object images^43^, on the analysis of diffraction rings (caused by Mie scattering)^36^ surrounding the object images, or on the evaluation of the intensity averaged particle radius^26^ in the images are known. Instead, however, we employ a metric based on the overall intensity gradient inside an image, known as the Tenenbaum gradient^44–46^ (TBG), which is an established measure for the sharpness of an image object and can be used for the development of autofocusing algorithms^44,45^. In an own separate study, metrics using the TBG and peak heights in intensity line profiles have been compared, where the TBG metric achieved the best *z*-height resolution (see Fig. S2.1 in the Supporting Information). Given an image of *M* x *N* pixels^2^, the TBG ${f}_{\mathrm{TBG}}$ sums over the squares of all *X* and *Y* image gradients ${G}_{x}\left(i,j\right)$ and ${G}_{y}\left(i,j\right)$ in pixels $\left(i,j\right)$^44^:

$${f}_{\mathrm{TBG}}=\sum_{i}^{M}\sum_{j}^{N}{G}_{x}(i,j{)}^{2}+{G}_{y}(i,j{)}^{2}$$

${G}_{x}\left(i,j\right)$ and ${G}_{y}\left(i,j\right)$ are convolutions of the image with Sobel operators^47^, which calculate an approximation of the image intensity gradient at a given position. For visualization, the convolved images ${G}_{x}\left(i,j\right)$ and ${G}_{y}\left(i,j\right)$ for the SPP containing images in Fig. 1d and e are shown in the Supporting Information (Fig. S3.1). To investigate whether ${f}_{\mathrm{TBG}}$ is a sensitive measure for relative particle height when investigating particles with different optical responses, it has been determined for focus sweeps of SPPs with two different prototypical compositions and with different diameters: micromer-M (*d* = 3 µm, 4 µm) and Dynabeads M-270 (*d* = 2.8 µm). While for micromer-M SPPs the superparamagnetic material is placed around a non-magnetic organic core^48^, Dynabeads M-270 SPPs consist of a homogenous distribution of superparamagnetic material throughout the whole particle^49^. Consequently, the optical responses of the investigated SPPs are different. In all cases, focus sweeps have been carried out by taking images after each step of 25 nm (50 nm for 4 µm SPP) in *z*-direction. TBG calibration curves have been obtained by optimizing the acquired microscope images of 800 pixels × 600 pixels and subtracting the inhomogeneous illumination background (for details see “Methods” section and S1 in the Supporting Information). SPPs within these images have been localized laterally using conventional two-dimensional single particle tracking software^50^. The center of the localized SPP has then been used to crop the image into regions of interest (ROIs), which were placed symmetrically around the center. The sizes of the ROIs ranged from 50 pixels × 50 pixels (corresponding to 5.5 µm × 5.5 µm) for 3 µm micromer-M and 2.8 µm Dynabeads M-270, and 60 pixels × 60 pixels (corresponding to 6.6 µm × 6.6 µm) for 4 µm micromer-M SPP, respectively.

**Figure 2:**
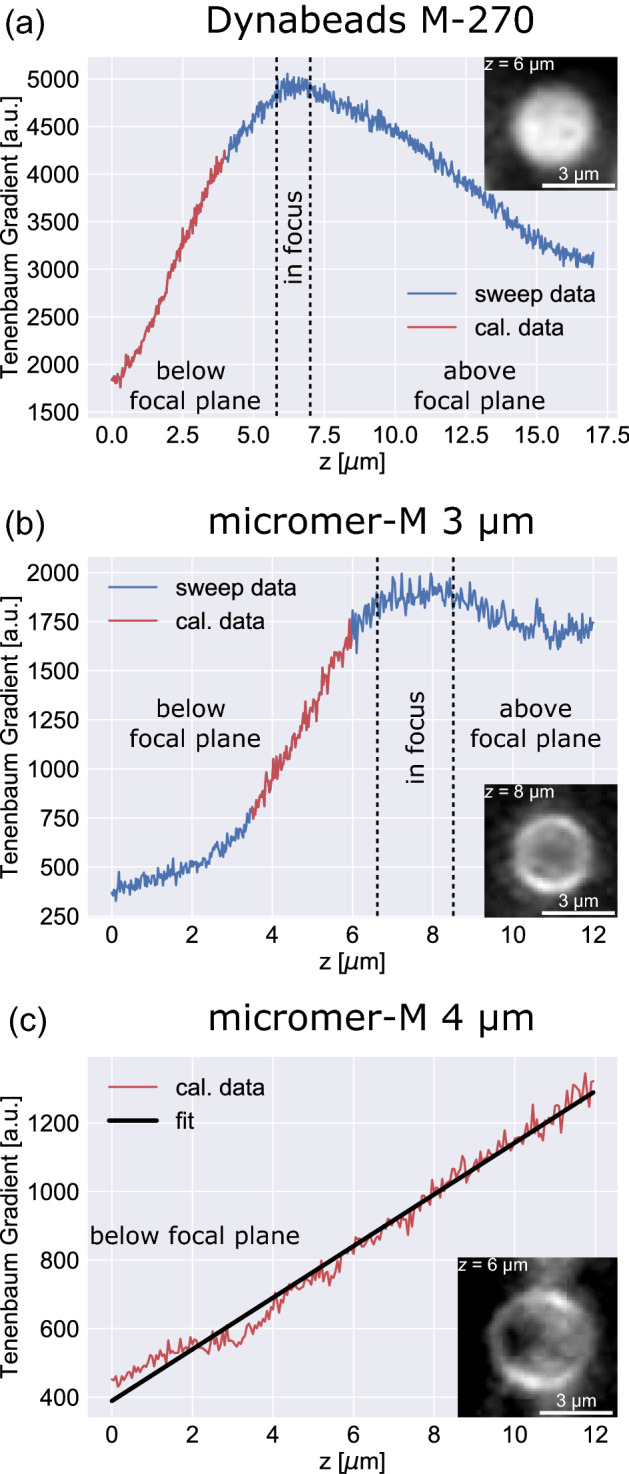
Exemplary focus sweep data for determining the *z*-position of SPPs within a microfluidic transport system. Curves (**a**–**c**) show the TBG, as a measure for particle sharpness, in dependence on the set *z*-position of the SPPs with respect to the focal plane. The data was acquired by sweeping the sample through the focal plane of the used microscope (starting with a position below the focal plane) in 25 nm steps for Dynabeads M-270/micromer-M 3 µm, and in 50 nm steps for micromer-M 4 µm, recording reference images at every step. Curves (**a**) and (**b**) show the general progression of TBG for different *z*-position regimes (below, within, and above the focal plane) and demonstrate that the chosen approach for evaluating the particle sharpness is suitable for different types of SPP. The regions of highest sensitivity (steepest slope) for *z*-position detection with approximately linear progression are highlighted in red. For micromer-M 4 µm SPP (**c**), only the linear regime of the highest sensitivity focus sweep data is displayed which was taken for further particle transport experiments as the *z*-position calibration. Exemplary background-subtracted images of the respective SPP at the indicated *z*-position are shown in the insets of each plot.

For each obtained ROI, ${f}_{\mathrm{TBG}}$ has been calculated along all recorded reference images representing a known *z*-position, which is plotted in Fig. 2a for an exemplary Dynabead M-270 SPP (2.8 µm) and in Fig. 2b for an exemplary micromer-M 3 µm SPP. Both data sets serve as a demonstration of the general ${f}_{\mathrm{TBG}}$ progression as a function of *z*-position for different position regimes (below, within, and above the focal plane), showing a maximum of the TBG for the *z*-position of the highest particle sharpness (see ROI of a particle in the insets of Fig. 2 plots). With *z*-positions above and below this reference point, the TBG decreases for both types of SPP. The data also indicates that for very high *z*-distances between particle position and focal plane, the TBG reaches a saturation state. This observation agrees well with previously reported investigations, prominently to be found in studies on autofocusing algorithms^44,45^. The present data, however, does not reflect a symmetric, Gaussian-like distribution of the TBG around the in focus position. For both particle types, the increasing flank is steeper than the decreasing one. All recorded data sets show that the chosen approach for determining the relative SPP position with respect to the microscope’s focal plane works, irrespective of the SPP size (for the chosen size range) and the composition.

Maximum sensitivity regarding changes in *z*-position is achieved by using the increasing branches of the focus sweep data. In this regime (highlighted in red in Fig. 2a,b), the TBG changes almost linearly with increasing *z*-position in a height range of about 2–4 µm. Consequently, particles have been placed at positions below the focal plane for the 3D analysis of the SPP transport motion in the following experiments. For an exemplary micromer-M 4 µm SPP, which was investigated in the transport experiment, the TBG focus sweep data is displayed in Fig. 2c for the linearly behaving regime with changing *z*-position. A linear fit of the data (black line) allows direct quantification of the *z*-position with respect to the focal plane when ${f}_{\mathrm{TBG}}$ has been determined from the individual frames of the transport experiment. For micromer-M 4 µm particles the averaged inversed linear slope of the fit functions has been determined to be 0.029 $\frac{\mathrm{\mu m}}{\mathrm{TBG unit}}$. It is important to note that the differing optical responses of the individual particles led to particle and position dependent slopes of the linear calibration fit functions. Consequently, the TBG calibration function shown in Fig. 2c is valid for the corresponding SPP at a chosen position in the field of view.

### 3D analysis of SPP transport

**Figure 3:**
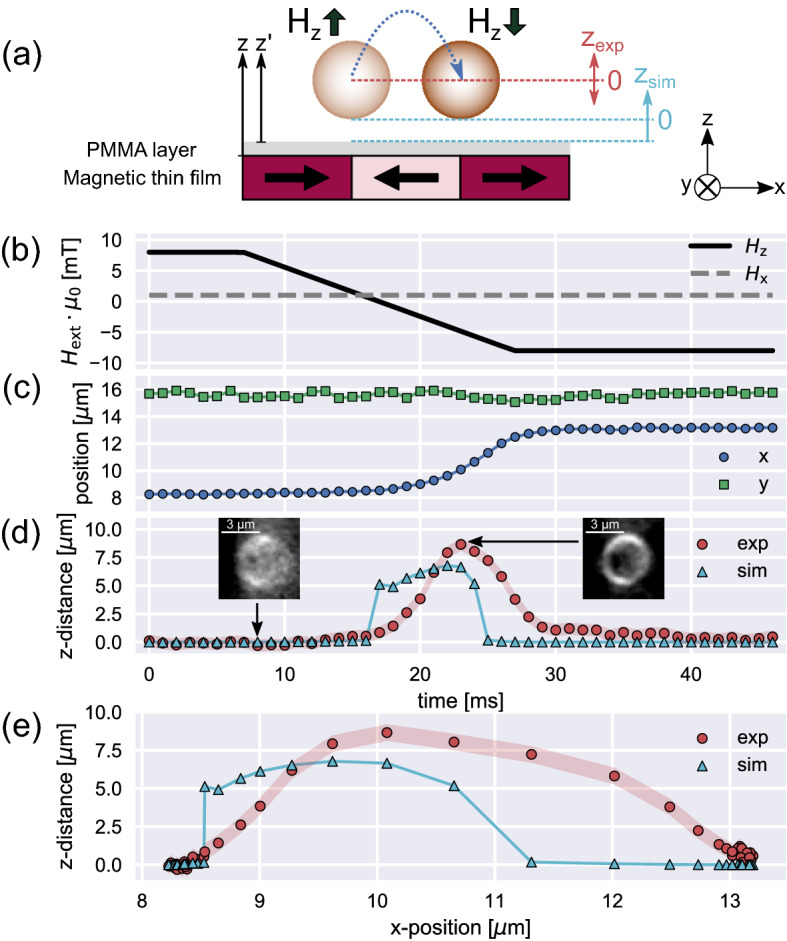
3D analysis of transport dynamics for 4 µm sized SPPs within a dynamically transformed MFL. (**a**) Sketch of one transport step for the SPP above a hh/tt magnetic stripe pattern (black arrows) after changing the external magnetic field in *z*-direction. The blue arrow indicates the expected “hopping” like motion of the SPP. Reference positions for the normalization of the experimentally determined vertical movement (*z*_exp_) and the theoretically estimated equilibrium distance (*z*_sim_) are indicated. Relevant forces for the estimation of *z*_sim_ were calculated along the indicated vertical axes z and z’ (see Supporting Information S5). (**b**) Applied external magnetic fields as a function of time for one pulse sequence. Black solid line: field in *z*-direction, grey dashed line: field in *x*-direction. (**c**) Plot of an exemplary SPP trajectory: determined *x*- (blue circles) and *y*- (green squares) SPP-center coordinates as functions of time. (**d**) Experimentally determined *z*-SPP center coordinates, relative to the equilibrium position of the SPP before the *z*-field change, superposed on the lateral movement (red circles). The initial *z*-position before the *z*-field change has been normalized to zero. Red shaded areas indicate the fit uncertainty for the used *z*-coordinate calibration function. For comparison, theoretical equilibrium distances between SPP and the substrate surface were computed (cyan triangles). The insets show background-subtracted microscopic images of the SPP at the indicated time. Changes in SPP sharpness and thereby *z*-position are observable. (**e**) Plot of determined *z*-positions in dependence on the *x*-positions for an SPP during a transport step. Again, red circles represent normalized experimental data (with the red shaded area as the uncertainty), while the cyan triangles depict simulated equilibrium distances. The data normalization was performed as described for (**d**). Data points of *x*-, *y*-position, and the simulated *z*-distance were connected to provide a guide to the eye.

Lateral transport of SPPs in the MFL over a magnetically stripe patterned substrate is inducible by periodic external magnetic field pulses in *x*- and *z*-directions^19,22,42^. The superposition of the static local stray fields and the external magnetic field transforms the potential energy landscape of SPPs in close vicinity to the substrate in such a way, that a stepwise movement can be forced^19^. In the present work, the behavior of micromer-M SPP (*d* = 4 µm) has been studied. One transport step towards the adjacent domain wall is inducible^19^ upon a switch of the external *z*-field from $+{H}_{ext,\mathrm{z},\mathrm{max}}$ to $-{H}_{ext,\mathrm{z},\mathrm{max}}$. This is schematically shown in Fig. 3a together with the expected 3D movement of an SPP: After a sign change in ${H}_{ext,\mathrm{z}}$, the magnetic force on the SPP is altered from attractive to repulsive, initially increasing the elevation of the SPP above the substrate.

As the SPP moves laterally in *x*-direction towards the new location of minimum potential energy, magnetic attraction increases, thus, forcing the SPP back to the vertical position it occupied prior to the transport step. For a test of the current 3D tracking strategy and to verify the true 3D particle motion experimentally, an external magnetic fields sequence was applied as shown in Fig. 3b. The field in *x*-direction has been kept constant at ${H}_{\mathrm{ext},\mathrm{x},\mathrm{max}}\cdot {\mu }_{0}= 1 \mathrm{mT}$. The *z*-field has been changed linearly from ${H}_{\mathrm{ext},\mathrm{z},\mathrm{max}}\cdot {\mu }_{0}= +8 \mathrm{mT}$ to ${H}_{\mathrm{ext},\mathrm{z},\mathrm{max}}\cdot {\mu }_{0}= - 8 \mathrm{mT}$ within 20 ms. This field change results in *x*- and *y*-trajectories, as presented for an exemplary SPP in Fig. 3c. Lateral *x*- and *y*-coordinates have been determined this time with a machine learning aided tracking method^29^ (see “Methods” section for further details). As expected, the *z*-field change leads to a step-like motion along the *x*-coordinate (blue circles in Fig. 3c) with the step width resembling the domain width of the magnetically patterned substrate. As the MFL does not change along *y*, the *y*-position (green squares in Fig. 3c) remains approximately constant apart from a small Brownian motion^19^.

From the microscope images, taken with a frame rate of 1000 fps, already qualitatively an axial *z*-movement can be identified during lateral SPP motion. This is illustrated by the microscope snapshots of an exemplary SPP in the insets of Fig. 3d. Prior to a *z*-field change, the SPP image shows low sharpness (and therefore a low ${f}_{\mathrm{TBG}}$), since the sample was placed below the microscope focal plane by the piezo stage. After the start of the lateral *x*-movement, a significant increase in sharpness/${f}_{\mathrm{TBG}}$ is observable. This corresponds to an upward movement of the SPP, as it is gradually elevated towards the focal plane of the microscope. For the quantitative analysis of the *z*-position, ${f}_{\mathrm{TBG}}$ was calculated for the cropped SPP in each recorded, background-subtracted frame and then turned into a *z*-coordinate using the individual calibration function for the respective particle. For the SPP in Fig. 3d (red circles), the particle is elevated up to approximately 8.7 µm above the initial *z*-position (taken as 0 for reference) upon an inversion of the external magnetic field in *z*-direction. As the SPP completes its lateral movement along *x*, the *z*-position decreases again back to the initial *z*-elevation.

## Discussion

The qualitative progression of the particle’s motion strongly resembles the theoretically predicted “hopping” like behavior^39^ (see Fig. 3a), which is underpinned by the tracked *z*-position in dependence on the *x*-position in Fig. 3e (red circles). Here, with the SPP moving from about 8 µm to about 13 µm, the *z*(*x*)-data exhibits an asymmetry with respect to the position of maximum elevation away from the substrate. The initial increase of the *z*-distance seems to be steeper than the following decrease back to the reference position. To compare the measured *z*(*x*) of SPPs within the lateral transport step with theoretical estimates, SPP-substrate distances have been calculated based on the equilibria of acting forces. Since the corresponding model is extensively described in previous works^23,25^, it will only be summarized briefly in the following, and the relevant equations and parameters will be given in the Supporting Information S5.

Attractive and repulsive forces, acting on the SPP along the *z*-axis, have been balanced for the calculation of an equilibrium distance between SPP and substrate at each time step (1 ms) during the transport experiment. The forces taken into account are the magnetic force ${F}_{\mathrm{M}}\left(z\right)$, the electrostatic force ${F}_{\mathrm{el}}\left(z{^{\prime}}\right)$, the van der Waals force ${F}_{\mathrm{vdW}}\left(z{^{\prime}}\right)$, and the effective gravitational force ${F}_{\mathrm{Grav}}$ (simultaneously considering gravity and buoyancy)^25^. As ${F}_{\mathrm{M}}$ for a SPP is determined by the derivative of its potential energy ${U}_{\mathrm{p}}$ within the MFL and consequently by the gradient of the effective magnetic field ${\overrightarrow{H}}_{\mathrm{eff}}={\overrightarrow{H}}_{\mathrm{ext}}+{\overrightarrow{H}}_{\mathrm{MFL}}$^25^, the superposition of the local static MFL ${\overrightarrow{H}}_{\mathrm{MFL}}$ and the externally applied field ${\overrightarrow{H}}_{\mathrm{ext}}$ needs to be calculated^23^. The *x-* and *z-*components of the static MFL ${H}_{\mathrm{MFL},x}(z,x)$ and ${H}_{\mathrm{MFL},z}(z,x)$ estimated from simulations are plotted in the Supporting Information S5. In previous studies^23,24^, the equilibrium distance between magnetic particle and substrate surface has been calculated for a position over a domain wall center in the underlying magnetic thin film system. For estimating the change of this distance upon lateral particle motion from one domain wall to the adjacent one, ${\overrightarrow{H}}_{\mathrm{eff}}$ needs to be computed as a function of the *x*-coordinate. This has been done by simulating the MFL of a parallel-stripe domain pattern, where ${\overrightarrow{H}}_{\mathrm{MFL}}$ has been derived for different *z*- and *x*-positions^23^ from results of micromagnetic simulations using the object oriented micromagnetic framework OOMMF^51^. ${H}_{\mathrm{MFL},z}\left(x,z\right)$ and ${H}_{\mathrm{MFL},x}\left(x,z\right)$ were subsequently approximated by an exponential decay function and a sixth-degree polynomial, respectively. The experimentally observed *x*-trajectory $x\left({t}_{i}\right)$ of the SPP has been used to determine ${F}_{\mathrm{M}}\left(z({x(t}_{i}))\right)$ at each time step ${t}_{i}$ based on the effective magnetic field ${H}_{\mathrm{MFL},z}(x({t}_{i}))+{H}_{\mathrm{ext},z}({t}_{i})$. Using this magnetic force, the force equilibrium leads to a corresponding equilibrium distance between SPP and substrate surface, which has been calculated as functions of ${t}_{i}$ and $x\left({t}_{i}\right)$. The results are displayed as cyan triangles in Fig. 3d and e, respectively, with the smallest calculated equilibrium distance set to zero for comparison.

The presented differences in the equilibrium distances are qualitatively comparable to the progression of the experimentally determined *z*-positions. In the calculations, the equilibrium distance increase and subsequent decrease appear at earlier times/smaller *x*-positions. The temporal increase and decrease of calculated equilibrium distances, moreover, emerges more abruptly compared to the experimental data. We stress that the estimated *x-*position-dependent equilibrium distances between substrate and SPP cannot fully reproduce the experimentally observed dynamic vertical motion, as the calculations do not consider inertial forces in the viscous medium due to friction^39^. Thus, for the chosen theoretical estimation of *z*-motion, the SPP jumps immediately upwards to the newly calculated equilibrium distance, as the previously attractive magnetic force ${F}_{\mathrm{M}}(z)$ is transformed into a repulsive force after the induced change in the external *z*-field. The differences between the present static force equilibrium model and reality will be more prominent for faster field switching. Figure 3 vividly shows the delayed *z*-motion due to the inertial force, but also demonstrates that the pulses used in the experiment are long enough that the equilibrium *z*-elevation is reached by the SPP in one transport step. The estimate predicts a maximum SPP elevation difference within one transport step of 6.8 µm, which is close to the experimentally determined maximum value of (8.7 ± 0.5) µm. For the investigated 4 µm micromer-M SPP, an uncertainty for the experimentally determined relative *z*-elevations of approximately 500 nm has been obtained (see Supporting Information S4 for further details). This uncertainty is indicated in Figs. 3d and e as shaded areas.

The results presented in Fig. 3 have been obtained for one exemplary SPP. As the individual TBG calibration function of one SPP may change with position due to the inhomogeneous lighting conditions, care has been taken whether the calibration function remains constant for the complete particle trajectory. A calibration function has been assumed to be not changing throughout the SPP motion when the TBG value for the SPP image measured before and after lateral movement is approximately equal. Under this condition, the *z*-movement of 4 additional particles could be quantitatively evaluated for the initiated transport step within the same experiment (trajectories are shown in Supporting Information S6). Averaging over all maximum height jumps, a value of (6 ± 2) µm has been obtained, being quantitatively comparable to the theoretically estimated maximum *z*-elevation of 6.8 µm. Here it should be noted that the used model tends to overestimate the acting magnetic force^23^, possibly resulting in overestimated *z*-distance changes. Differences in experimentally determined SPP *z*-elevations may be due to variations in the local MFL, the thickness of the deposited PMMA layer, and differences in the actual size and susceptibility of the investigated SPP.

## Conclusion

In this work, we presented a fully quantitative 3D analysis of SPP trajectories moved by a dynamically transformed MFL. This MFL emerges from a prototypical exchange-biased magnetic thin film substrate with engineered 5 µm wide magnetic hh/tt parallel-stripe domains. A surface-near lateral transport of SPP in a microfluidic environment was induced by the superposition with time-dependent external magnetic fields. The non-trivial quantitative determination of the axial *z*-movement has been achieved by analyzing the particle sharpness in microscope images when the SPP are observed at different distances to the focal plane in combination with the automatic acquisition of reference images during a focus sweep. The TBG for cropped SPP images as a measure of image sharpness was shown to be a powerful metric for *z*-coordinate determination in this type of experiment. A specific TBG, therefore, correlates to a defined *z*-position of the SPP. The validity and applicability of this approach have been demonstrated for two different types of SPP with different optical responses: micromer-M and Dynabeads M-270. With this technique, a frame-by-frame evaluation of 1000 fps videos from SPPs moving on 3D trajectories was possible. The theoretically expected “hopping” like motion of SPPs, crossing a stripe domain to reach the adjacent potential energy minimum above a neighboring domain wall, could be proven unequivocally. Using individual calibration functions, the maximum average vertical elevation of the investigated SPP was quantified to be (6 ± 2) µm for micromer-M SPP with *d* = 4 µm, as compared to a theoretically estimated value of around 6.8 µm. The findings pave the way for an in-depth understanding of magnetic particle dynamics within tailored magnetic stray fields, especially for application in LOC systems. The presented method is label-free and capable to be applied in high as well as standard frame rate video sequences. The quantifiable movement along the *z*-axis will open a new way for the detection of analytes in diagnostic LOC devices by optical bright-field microscopy and for material analysis of surfaces covered by liquids: As the interactions between SPP and surface will be modified upon either particle surface or substrate surface coverage with analytes, the equilibrium distances between SPP and substrate will change accordingly. With the presented method, these changes can be quantified, indicating the presence of an analyte. Similarly, surface material differences, local varieties in the MFL, or even more general differences in the acting forces may be quantified, possibly observing many particle heights and trajectories simultaneously.

## Methods

### Fabrication of magnetically patterned substrate

The prototypically used magnetic stripe domain pattern was imprinted into an exchange-biased magnetic thin film system by applying IBMP^21,30,31^. The thin film system consists of a Cu^10 nm^/Ir_17_Mn_83_^30 nm^/Co_70_Fe_30_^10 nm^/Au^10 nm^ layer system deposited onto naturally oxidized Si (100) via rf-sputtering at room temperature. Field cooling initialized the in-plane direction of the EB by annealing in a vacuum chamber (base pressure = 5 × 10^–7^ mbar) at 300 °C for 60 min in an in-plane magnetic field of 145 mT. For IBMP, a photoresist, that was thick enough to prevent 10 keV He ions from penetrating the magnetic layer system, was spin-coated onto the sample and 5 µm wide stripe structures (periodicity of 10 µm) perpendicular to the initial EB direction were fabricated by photolithography (Karl Suss MA-4 Mask Aligner). Subsequently, a home-built Penning ion source was used to bombard the sample with He ions at a kinetic energy of 10 keV (ion dose = 1 × 10^15^ cm^−2^). To obtain a hh/tt magnetic domain configuration within the thin film system, a homogenous magnetic field (100 mT), antiparallel to the initial EB direction, was applied in-plane during ion bombardment. After bombardment, removal of the photoresist was achieved by treating the sample successively in an ultrasonic bath for 5 min at 50 °C in a 3% KOH solution and for 3 min at 50 °C in acetone and water. The sample was cleaned with acetone, isopropanol, and water and dried in a N_2_ stream. Finally, a 150 nm thick PMMA layer was deposited on top of the sample by spin-coating.

### Focus sweeps and particle transport

For the calibration of the *z*-coordinate for SPP, a transport substrate with SPP residing on top was swept in discrete steps through the focus plane of the used microscope. Investigated SPP were obtained from micromod Partikeltechnologie GmbH (micromer-M) and Thermo Fisher Scientific Inc. (Dynabeads M-270 Carboxylic Acid). Beforehand, 20 µl of a diluted SPP dispersion in water was pipetted into a microfluidic chamber on top of the transport substrate. The chamber was fabricated by cutting a ca. 5 mm × 5 mm sized window into a square sheet of Parafilm and attaching the resulting structure to the substrate. After SPP deposition, the chamber was sealed with a cover glass (00 strength) and a drop of immersion oil (AppliChem GmbH, A0699,0100) was placed onto it. Subsequently, calibration reference images (800 pixels × 600 pixels) were recorded by using an optical bright-field microscope with a 100 × magnification objective (Nikon, N.A. = 1.4) and a high speed camera (Optronis CR450 × 2). For this purpose, the sample was moved axially in 25 nm steps (3 µm SPP) or 50 nm steps (4 µm SPP) via a Piezo stage (P-603.3S2 connected to PI E-625 PZT Servo Controller) and after each step, an image was taken. Directly after the sweep, transport experiments were performed using a home-built setup consisting of electromagnets for the application of homogenous magnetic fields in *x*- and *z*-direction. The sample had been placed into the setup with its plane being parallel to the direction of the magnetic field generated by the *x*-coils. Hence, the magnetic field in *z*-direction was perpendicular to the substrate plane. The magnetic stripe domains long axis was aligned perpendicular to the *x*-direction. Controlled particle motion was induced by applying a time-dependent field in the *z*-direction as sketched in Fig. 3b. Videos of the particles’ motion from one to the next domain wall were recorded with the same camera using a 1000 fps frame rate.

### Particle tracking

Lateral SPP positions in *x*- and *y*-direction were tracked for focus sweep images using the Video Spot Tracker software^50^ and for transport video recordings using the Python-based program AdaPT, which is specifically designed for the analysis of spherical magnetic particles^29^. For the latter, particle locations are identified in each frame using an intensity-based method. Machine learning techniques are employed to derive optimal parameters for the localization algorithm. Subsequently, particle trajectories are obtained via a frame-by-frame linking procedure. The found *x*- and *y*-positions of the SPP are used to isolate each tracked particle for the *z*-coordinate measurements. Prior to that, raw images were contrast-enhanced through histogram equalization and background noise was reduced by applying low-pass filtering to the Fourier transform of the images with a following inverse Fourier transformation conducted. A subsequent subtraction of the inhomogeneous illumination background was done by averaging over all images of the SPP transport videos and then afterwards subtracting the obtained average image from all background-including images (focus sweep and transport). The background subtraction is a required preprocessing step for the images from subsequently performed SPP transport experiments: When particles change their lateral position, they can be exposed to different lighting conditions along their trajectory. Here, the background subtraction minimizes the illumination dependent changes of ${f}_{\mathrm{TBG}}$ which finally allows using the calibration data. In the processed images, ROIs sized 50 pixels × 50 pixels (3 µm SPP) and 60 pixels × 60 pixels (4 µm SPP) were cropped around each SPP, with the particle being centered. The TBG was computed for the ROI in each time frame of the recorded videos and *z*-coordinate identification was completed by using the respective, particle-specific calibration function from the focus sweep data.

## Supplementary Information

Supplementary Information.

## Data Availability

Original data will be made available by the corresponding authors upon reasonable request.

## Abbreviations

LOC: Lab-on-a-chip
MP: magnetic particle
MFL: magnetic field landscape
3D: three-dimensional
SPP: superparamagnetic particle
hh: head-to-head
tt: tail-to-tail
EB: Exchange-bias
IBMP: ion bombardment induced magnetic patterning
PMMA: Poly(methyl methacrylate)
DOF: depth of field
TBG: Tenenbaum gradient
ROI: region of interest
