# Supplementary material for: Three-dimensional close-to-substrate trajectories of magnetic microparticles in dynamically changing magnetic field landscapes
Source: Sci Rep. 2022 Dec 3;12:20890. doi: 10.1038/s41598-022-25391-z (PMC9719552; doi:10.1038/s41598-022-25391-z)
Supplement: Supplementary file 1 — Supplementary Information. [file 41598_2022_25391_MOESM1_ESM.pdf]

Three-dimensional close-to-substrate trajectories of magnetic microparticles in dynamically changing magnetic field landscapes

Rico Huhnstock, Meike Reginka, Claudius Sonntag, Maximilian Merkel, Kristina Dingel, Bernhard Sick, Michael Vogel, Arno Ehresmann**

E-Mails: rico.huhnstock@physik.uni-kassel.de; ehresmann@physik.uni-kassel.de

Supporting Information

List of contents:

S1 Image processing

S2 Comparison of metrics

S3 Application of Sobel operator

S4 Estimation of *z*-coordinate uncertainty

S5 Theoretical model: important equations & parameters

S6 Vertical trajectories for all evaluable particles

S1 Image processing

To optimize the microscope images before further evaluation, they were contrast-enhanced and noise-reduced. For contrast enhancement, the histogram equalization function from the "scikits-image" Python library was applied[1]. Subsequently, noise in the images was reduced by performing a Fast Fourier Transformation (FFT), using the respective function from the Python-based "Numpy" library[2], then applying a home-programmed low-pass filter, and finally performing an inverse FFT. The image data were normalized to exhibit a minimum pixel value of 0 and a maximum of 255 using the Python "Open-CV" library[3]. For comparison, raw image (a), contrast-enhanced image (b), and noise-reduced images (c) of an exemplary video frame showing several SPPs are shown in Figure S1.1.

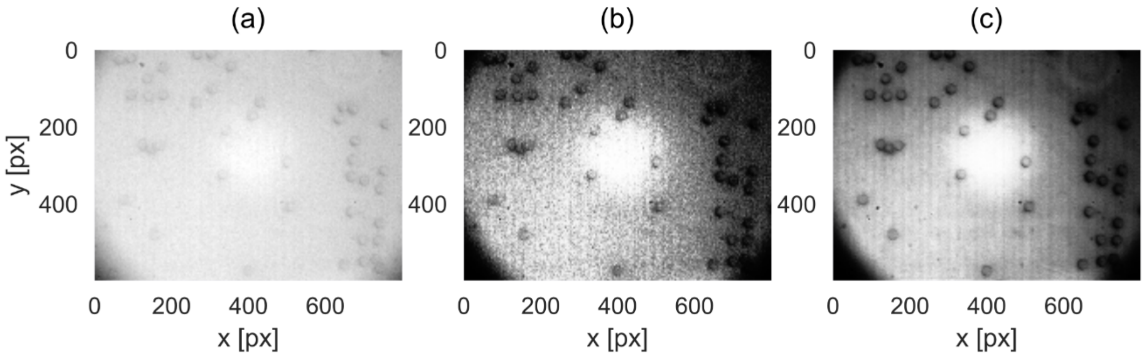

Figure S1. 1: Display of raw (a), contrast-enhanced (b), and noise-reduced (c) images for SPP.

S2 Comparison of metrics

To employ the most suitable metric for the identification of SPP *z*-coordinates in the image data derived from the described experiments, the Tenenbaum gradient (TBG) and line profile peak heights for cropped SPP images were compared. The latter was determined by extracting a line profile across the SPP image and then calculating the peak height observable at the position of the particle edge. The results are displayed in Figure S2.1, deeming the TBG more suitable, as the lower data noise provides higher *z*-coordinate accuracy.

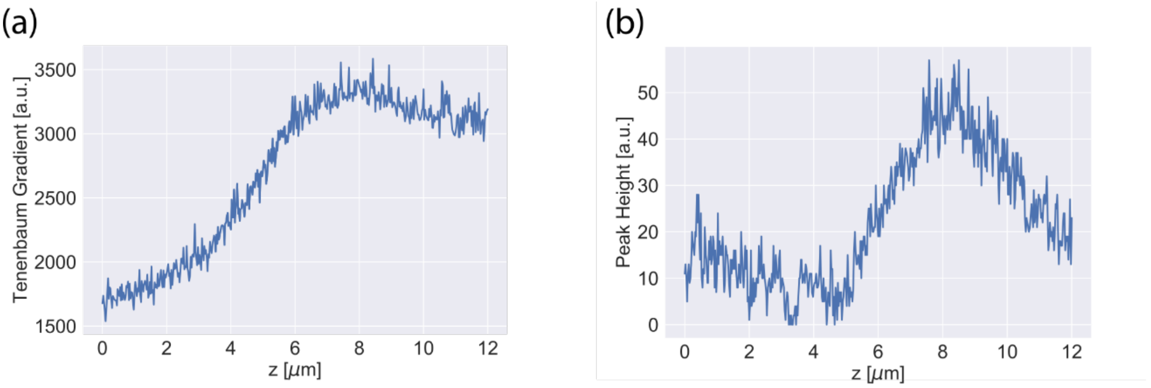

Figure S2. 1: Plots of TBG (a) and intensity line profile peak height (b) in dependence on the axial z-coordinate for non-background subtracted images of a micromer-M SPP with d = 3 µm.

S3 Application of Sobel operator

For the calculation of the TBG, Sobel operators were applied to the optimized images in *x*-(horizontal) and *y*-direction (vertical) using the Python-based “Open-CV” library[3]. Exemplary results of these operations are shown in Figure S3.1.

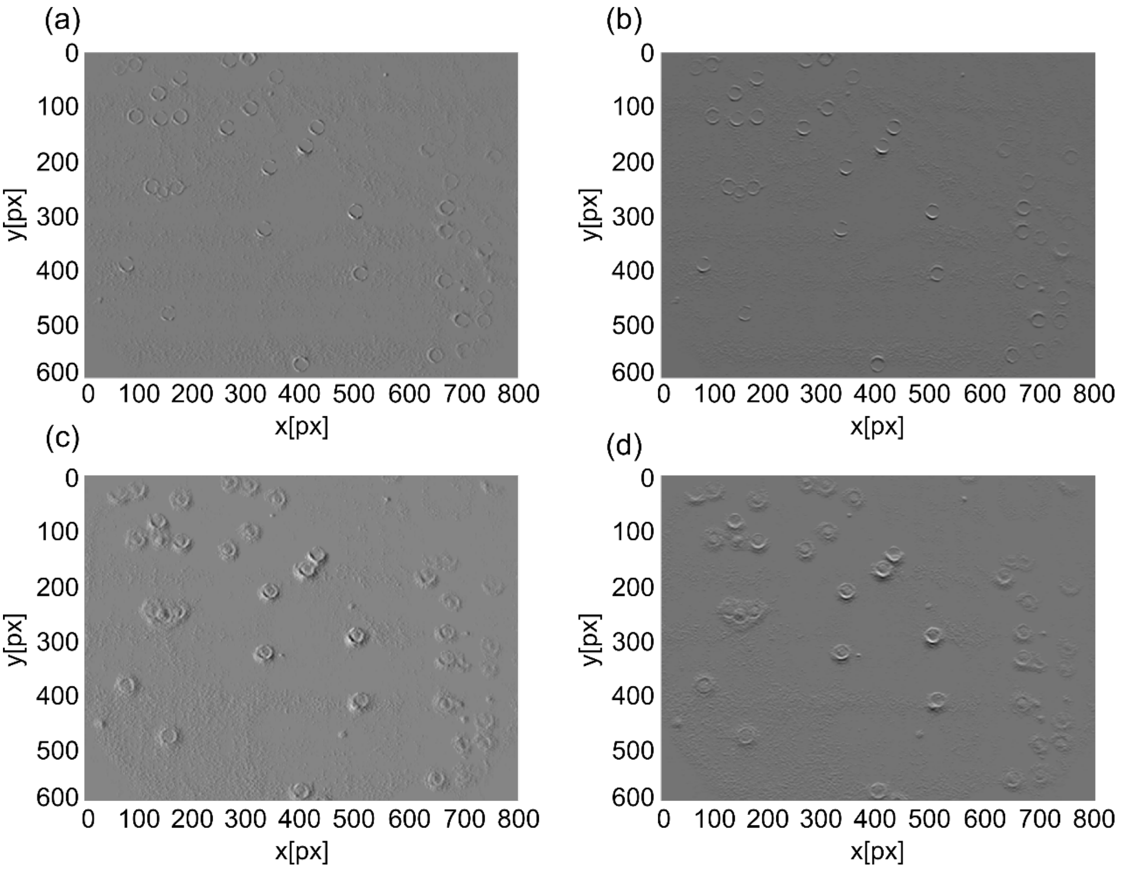

Figure S3. 1: Convolution of optimized images with Sobel operators. (a) and (b) display the convolved images for focussed SPP in x- and y-direction, respectively, while (c) and (d) display the convolved images for SPP above the focal plane.

S4 Estimate of *z*-position determination uncertainty

Some experimental conditions need to be considered when assessing the presented 3D tracking of SPP in dynamically transformed MFL. First, the accuracy of the employed *z*-coordinate calibration procedure is mainly influenced by the recorded image noise and therefore the quality of the linear fit to the focus sweep data. It was found that background subtraction significantly reduces the noise in the calculated TBG as a function of the *z*-coordinate (see S1). Additionally, it diminishes artifacts due to inhomogeneous illumination during the experiment. The uncertainty for z-coordinate determination from the acquired fit function was calculated by considering the mean residual of experimental focus sweep data z_{sweep} and linear fit $z_{\text{fit}}(f_{\text{TBG}})$:

$$\text{mean residual} = \frac{\sum_i^N \left| z_{\text{sweep,i}} - z_{\text{fit}}\left(f_{\text{TBG,i}}\right) \right|}{N},$$

with the number of sampling points *N*. Other sources of uncertainty include the accuracy of axial positioning during the focus sweep by the installed piezo stage, focus drifts during focus sweep and transport image data acquisition, and unintended movement of the sample plane due to, e.g., external vibrations. As these influences are either negligible or not quantifiable, we take the above-described measure for the error value of the determined SPP *z*-positions in the transport videos.

S5 Theoretical model: important equations & parameters

According to the theoretical model proposed in Ref.[4], equilibrium elevations above the flat substrate for micromer-M SPP (*d* = 4 µm) within a periodic magnetic field landscape (MFL) were calculated by balancing the vertically directed gravitational, buoyancy, magnetic, electrostatic, and van-der-Waals forces. The used formulae and parameters for the calculations are presented in the following:

<u>Gravitational force:</u>

$$F_{\mathrm{G}} = \frac{4}{3} \cdot \pi \cdot r^3 \cdot \rho_{\mathrm{particle}} \cdot g$$

r: particle radius

ρ_{particle}: particle density

g: gravitational acceleration

<u>Buoyancy force:</u>

$$F_{\mathrm{B}} = \frac{4}{3} \cdot \pi \cdot r^3 \cdot \rho_{\mathrm{liquid}} \cdot g$$

ρ_{liquid}: liquid density

<u>Magnetic force[5]:</u>

$$\vec{F}_{\mathrm{m}}(x,z) = -\mu_0 \cdot \left(\vec{m}_{\mathrm{b}}(x,z) \cdot \vec{\nabla}\right) \cdot \vec{H}_{\mathrm{eff}}(x,z)$$

μ_0: vacuum permeability

$\vec{H}_{\mathrm{eff}}(x,z)$: superposition of external field $\vec{H}_{\mathrm{ext}}$ and MFL $\vec{H}_{\mathrm{MFL}}$

$\vec{m}_{\mathrm{b}}(x,z)$: magnetic moment of particle calculated by Langevin function[6]:

$$\vec{m}_{\mathrm{b}}(x,z) = m_{\mathrm{s}} \cdot \left[\coth\left(b \cdot \vec{H}_{\mathrm{eff}}(x,z)\right) - \left(\frac{1}{b \cdot \vec{H}_{\mathrm{eff}}(x,z)}\right)\right]$$

m_{s}: saturation magnetic moment

b: Langevin parameter

<u>Electrostatic force[7]:</u>

$$F_{\mathrm{el}}(z') = \frac{2 \cdot \pi \cdot \varepsilon_r \cdot \varepsilon_0 \cdot r \cdot \kappa}{1 - e^{-2\kappa z'}} \cdot \begin{bmatrix} 2 \cdot \zeta_{\mathrm{particle}} \cdot \zeta_{\mathrm{substrate}} \cdot e^{-\kappa z'} \\ \pm\left(\zeta_{\mathrm{particle}}{}^2 + \zeta_{\mathrm{substrate}}{}^2\right) \cdot e^{-2\kappa z'} \end{bmatrix}$$

$\varepsilon_r \varepsilon_0$: permittivity of the liquid

κ: inverse Debye length

$\zeta_{\mathrm{particle}}$: particle zeta potential

$\zeta_{\mathrm{substrate}}$: substrate zeta potential

<u>Van der Waals force[8]:</u>

$$F_{\mathrm{vdW}}(z') = \frac{A_{\mathrm{H,123}} \cdot r}{6 \cdot z'^2}$$

$A_{\mathrm{H,123}}$: Hamaker constant for particle(1)/substrate(2)/liquid(3) system

Particle radius *r*	2 µm
Particle density ρ_{particle}[9]	1300 kg/m³
Particle saturation magnetic moment m_{s}[5]	$4.48 \cdot 10^{-14}\ \mathrm{Am}^2$
Langevin parameter *b*[5]	$1.05 \cdot 10^{-4}\ \mathrm{m}/\mathrm{A}$
Particle zeta potential $\zeta_{\mathrm{particle}}$[9]	-32 mV
Substrate zeta potential $\zeta_{\mathrm{substrate}}$[10]	-65 mV
Thickness resist layer t_{resist}	150 nm
Thickness capping layer t_{cap}	10 nm
Debye length 1/κ[11]	100 nm
Hamaker constant $A_{\mathrm{H,123}}$[12]	$1.23 \cdot 10^{-20}\ \mathrm{J}$
Liquid density ρ_{liquid}	1000 kg/m³

Table S5. 1: Used parameters for the calculation of relevant forces determining the steady-state distance between the particle and the underlying substrate.

The MFL was computed by simulating the employed hh/tt magnetization pattern within the ferromagnetic $Co_{70}Fe_{30}$ layer, using the object-oriented micromagnetic framework (OOMMF)[13]. Here, a grid of *x* = 20 µm and *y* = 10 µm and a volume of (5 nm)^3 for the cubic mesh elements within this grid was implemented. The EB related unidirectional anisotropy within each stripe domain was considered as a fixed Zeeman term with an alternating sign for differently magnetized stripes. The magnitude of the Zeeman term was chosen to be the EB field of the respective stripe domain type measured by magneto-optical Kerr magnetometry[4]. Due to the fabrication process of the domain pattern (ion bombardment induced magnetic patterning), saturation magnetization[14] and uniaxial anisotropy[15] within the bombarded stripes are decreased and values for the simulation were chosen accordingly[4]. Once the micromagnetic computation has converged, the magnetic moment for each mesh element was calculated by multiplying the obtained magnetization in the *x*-direction with the mesh element volume. The MFL at position $\vec{r}(x, y, z)$ consisting of the individual components $\vec{H}_x(\vec{r})$ and $\vec{H}_z(\vec{r})$ was then calculated according to the dipole approximation[16]:

$$\vec{H}(\vec{r}) = \frac{1}{4\pi} \cdot \sum_i \frac{3 \cdot \left(\vec{R} \cdot \vec{m}_i\right) \cdot \vec{R}}{\left|\vec{R}\right|^5} - \frac{\vec{m}_i}{\left|\vec{R}\right|^3}.$$

$\vec{R} = \vec{r} - \vec{r}_i$ represents the distance vector between position $\vec{r}$ and dipole position (mesh element position) $\vec{r}_i$. For visualization, the *z*- and *x*-component of the MFL are plotted in Figure S5.1 in dependence on the *x*- and *z*-coordinates above the substrate surface.

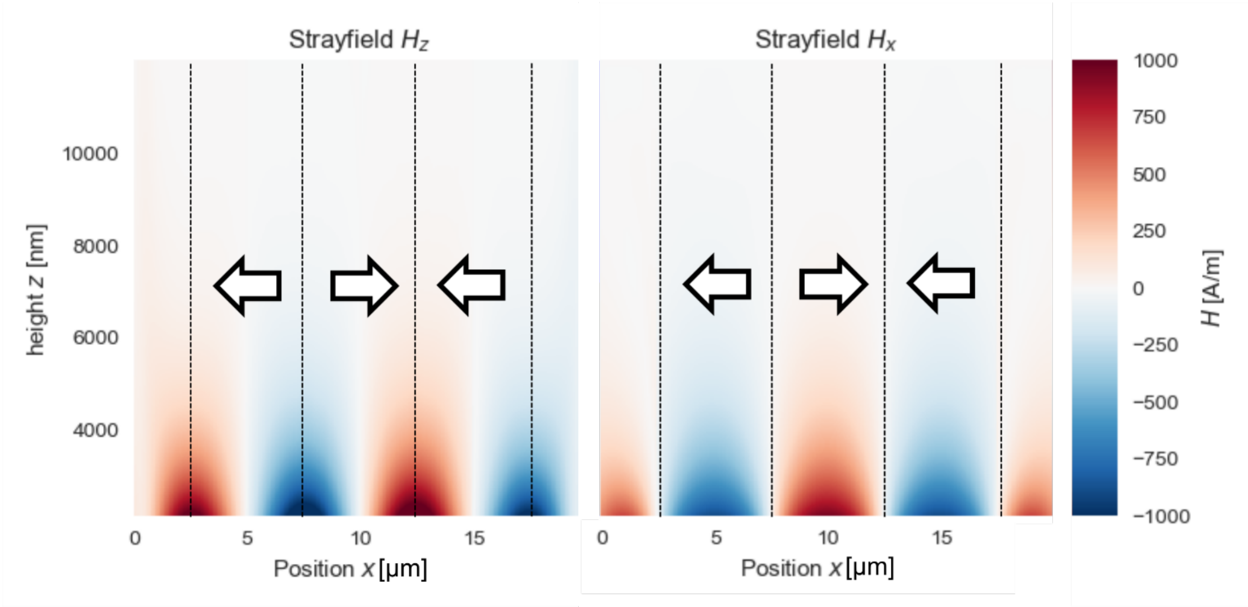

Figure S5.1: Distribution of magnetic stray fields in *x*- and *z*-direction on top of magnetically stripe domain patterned substrates with head-to-head/tail-to-tail configuration (as indicated by arrows). The magnetic field strength is color-coded. The *z*-dependent progression of both H_z and H_x results from fitting an exponential decay and a polynomial function, respectively, to distinct values of $H_{z,x}$ calculated from a magnetization pattern at a set of *z*-heights. The pattern was derived from micromagnetic OOMMF simulations.

S6 Vertical trajectories for all evaluable particles

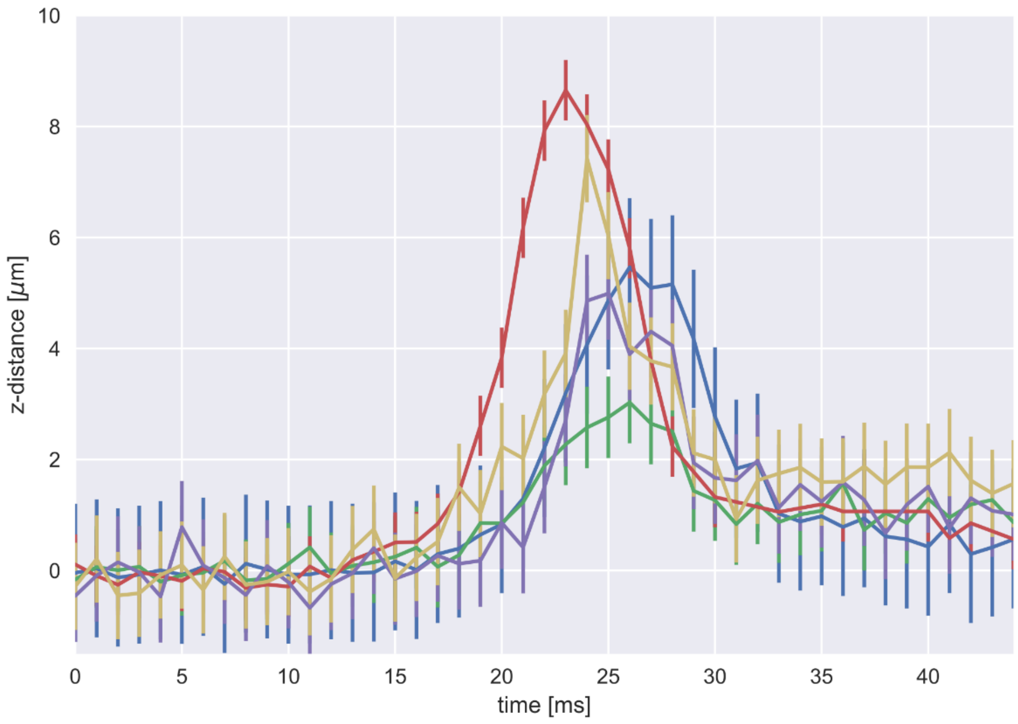

Figure S6.1: Experimentally measured vertical *z*-trajectories of five SPPs in the analyzed particle transport experiment. Error bars are indicating the uncertainty of the respectively used calibration function for *z*. Note that all "jumps" in the displayed data might not occur at the same time, since some frames were lost during conventional two-dimensional particle position tracking.

1. Van Der Walt, S. *et al.* Scikit-image: Image processing in python. *PeerJ* **2014**, 1–18 (2014).
2. Harris, C. R. *et al.* Array programming with NumPy. *Nature* **585**, 357–362 (2020).
3. Bradski, G. The OpenCV Library. *Dr. Dobb's J. Softw. Tools* (2000).
4. Reginka, M. *et al.* Transport Efficiency of Biofunctionalized Magnetic Particles Tailored by Surfactant Concentration. *Langmuir* **37**, 8498–8507 (2021).

5. Holzinger, D., Koch, I., Burgard, S. & Ehresmann, A. Directed Magnetic Particle Transport above Artificial Magnetic Domains Due to Dynamic Magnetic Potential Energy Landscape Transformation. *ACS Nano* **9**, 7323–7331 (2015).

6. Yoon, M. *et al.* Superparamagnetism of transition metal nanoparticles in conducting polymer film. in *Journal of Magnetism and Magnetic Materials* **272**–**276**, E1259–E1261 (2004).

7. Wirix-Speetjens, R., Fyen, W., Xu, K., De Boeck, J. & Borghs, G. A force study of on-chip magnetic particle transport based on tapered conductors. in *IEEE Transactions on Magnetics* **41**, 4128–4133 (2005).

8. Gregory, J. Approximate expressions for retarded van der waals interaction. *J. Colloid Interface Sci.* **83**, 138–145 (1981).

9. micromod Partikeltechnologie GmbH. Technisches Datenblatt: micromer-M, PEG-COOH, 4 µm.

10. Khademi, M., Wang, W., Reitinger, W. & Barz, D. P. J. Zeta Potential of Poly(methyl methacrylate) (PMMA) in Contact with Aqueous Electrolyte–Surfactant Solutions. *Langmuir* **33**, 10473–10482 (2017).

11. Butt, H., Graf, K. & Kappl, M. *Physics and Chemistry of Interfaces*. Wiley (2003).

12. Feldman, K., Tervoort, T., Smith, P. & Spencer, N. D. Toward a force spectroscopy of polymer surfaces. *Langmuir* **14**, 372–378 (1998).

13. Donahue, M. J. & Porter, D. G. OOMMF User's Guide, Version 1.0. *Natl. Inst. Stand. Technol. Gaithersburg, MD* (1999).

14. Huckfeldt, H. *et al.* Modification of the saturation magnetization of exchange bias thin film systems upon light-ion bombardment. *J. Phys.*

Condens. Matter **29**, 125801 (2017).

15. Müglich, N. D. *et al.* Preferential weakening of rotational magnetic anisotropy by keV-He ion bombardment in polycrystalline exchange bias layer systems. *New J. Phys.* **20**, 053018 (2018).

16. Nolting, W. Grundkurs Theoretische Physik 3. Springer (2013).
